# Pre-T cell receptor for improved expansion of TCR alpha disrupted T cells

**DOI:** 10.1186/2051-1426-1-S1-P9

**Published:** 2013-11-07

**Authors:** Roman Galetto, Céline Lebuhotel, Laurent Poirot, Cécile Schiffer-Mannioui, Sylvain Arnould, Julianne Smith, Andrew Scharenberg

**Affiliations:** 1 Cellectis Therapeutics, Paris, France

## 

Recent data have emerged from adoptive T-cell therapies where exogenous expression of a chimeric antigen receptor (CAR) has been shown to confer cancer recognition on autologous T cells. However, the ability to apply this technology in an allogeneic setting would permit the generation of universal “off the shelf” T cells that would overcome many current technical and logistic hurdles to the practical application of adoptive immunotherapies. Transcription Activator-Like Effector Nucleases (TALEN^TM^) can be used to inactivate the T cell receptor (TCR) alpha gene, eliminating the TCR and thus the potential of graft versus host disease (GVHD), one of the major obstacles towards an allogeneic approach. However, TCR disruption also results in the elimination of the CD3 signaling complex from the T-cell surface, and thus may alter the cells’ capacity for expansion and/or survival. The pre-T cell receptor (pre-TCR) is expressed by immature thymocytes and is crucial for T cell development. Pre-TCR consists of an invariant pre-T alpha chain, variable rearranged TCR beta chains and CD3 signaling components. In contrast to the TCR, which requires interaction with peptide-loaded major histocompatibility complexes to initiate T cell signaling, the pre-TCR is thought to signal through a ligand-independent mechanism that occludes TCR surfaces required for MHC interaction. Here we demonstrate that the expression of the invariant pre-T alpha chain, in the absence of TCR alpha, results in the restoration of CD3 at the cell surface in association with a pre-TCR. Cells with preTCR/CD3 complexes have an improved life span, and can be expanded ex vivo through standard CD3/CD28-based bead methods. Application of this technology in association with allogeneic CAR modified T cells will also be presented.

